# Knowledge and attitude toward HIV pre-exposure prophylaxis among women of reproductive age in Tanzania: Insights from the 2022 Tanzania health demographic survey

**DOI:** 10.1097/MD.0000000000043420

**Published:** 2026-09-25

**Authors:** Patrick Mwanahapa, Elihuruma Eliufoo Stephano, Aidat Mugula, Mtoro J. Mtoro

**Affiliations:** a Department of Public Health and Health Sciences, Nexus International University, Kampala, Uganda; b Department of Clinical Nursing, School of Nursing and Public Health, University of Dodoma, Dodoma, Tanzania; c Independent Consultant, Dar es Salaam Tanzania; d Global Programs, Dar es Salaam, Tanzania.

**Keywords:** attitude, HIV, knowledge, pre-exposure prophylaxis, reproductive age, Tanzania, women

## Abstract

Pre-exposure prophylaxis (PrEP) is an antiretroviral medication used to prevent human immunodeficiency virus (HIV) infection. Owing to the high incidence and prevalence of HIV Infection among women compared to men, PrEP has the potential to reduce the rate of HIV infection among women in sub-Saharan Africa, including Tanzania. This study aimed to assess women’s knowledge of and attitudes toward PrEP and its associated factors as an effective strategy for HIV prevention, using nationwide data. This study utilized data from the 2022 Tanzania demographic and health survey conducted between February and July 2022, the stratified sampling, by geographic region and urban/rural areas, employed a 2-stage process: primary sampling units from census areas, then households via probability systematic sampling. STATA 18.5 was used for data management and analysis, including descriptive statistics and weighted logistic regression to identify factors associated with PrEP knowledge and attitudes. Odds ratios (OR) and 95% confidence intervals (CIs) were used to estimate the strength of association. A total of 15,254 women with a mean age of 29.3 years (standard deviation = 9.8) were included in the study. The study found that 5.6% of women had knowledge of and positive attitudes towards HIV PrEP. In the adjusted analysis, those aged 35 to 49 years (adjusted odds ratio [AOR] = 2.09, 95% CI = 1.52–2.88), women with media exposure (AOR = 1.30, 95% CI = 1.24–1.83), women who had visited a health facility in the past 12 months (AOR = 1.44, 95% CI = 1.18–1.75), women who had ever heard of sexually transmitted infections (AOR = 1.51, 95% CI = 1.11–2.04), and those who had ever been tested for HIV (AOR = 1.48, 95% CI = 1.02–2.15) were significantly associated with knowledge and attitude towards HIV PrEP among women of reproductive age in Tanzania. This study found low knowledge of and attitude toward PrEP, highlighting the importance of PrEP education in women of reproductive age. However, strengthening PrEP education in health facilities’ service entry points, expanding educational initiatives through strategic community outreach programs, and media engagement represent transformative approaches to improving knowledge and attitude.

## 1. Introduction

Pre-exposure prophylaxis (PrEP) is a promising biomedical human immunodeficiency virus (HIV) prevention strategy that offers hope to reduce new HIV infections for those at substantial risk of HIV infection. PrEP protects the immune system from HIV infection through either a once-daily oral pill, which is globally available and widely used, a vaginal ring (dapivirine), designed specifically for women, and long-acting injectable PrEP both of which effectively prevent HIV acquisition by creating a protective barrier.^[1–3]^ The development of PrEP began in 2010 when women participating in clinical trials demonstrated the effectiveness of a 1% tenofovir vaginal gel for HIV prevention, followed by successful efficacy trials of oral tenofovir/emtricitabine in men.^[4]^ These initial findings were subsequently reinforced by additional studies confirming that oral PrEP containing tenofovir provides effective protection against HIV infection^[4,5]^ leading to the official approval of PrEP as an HIV prevention strategy in 2012.^[3]^

In 2015, the World Health Organization (WHO) conducted a meta-analysis of clinical trials and an open-label extension study, leading to a strong recommendation for oral PrEP containing tenofovir for all individuals at a significant risk of HIV infection, regardless of sex or demographic category.^[6]^ Tanzania began implementing PrEP in 2018 for populations at substantial HIV risk. Initial demonstration projects ran from May 2018 to March 2019 across 34 councils in the Ruvuma, Iringa, Mbeya, Njombe, Dar es Salaam, Mwanza, Kagera, Shinyanga, and Songwe regions.^[7]^ Despite this rollout, by 2022/2023, Tanzania showed a notable gender disparity in HIV rates, with women experiencing both a higher incidence (0.24% vs 0.11%) and prevalence (5.6% vs 3.0%) than men.^[8]^ Yet women’s utilization of PrEP remains surprisingly low, even though it offers a private, user-controlled method that allows women to protect themselves independently of their partners’ actions.^[9]^ PrEP is an evidence-based intervention for HIV transmission prevention, as it significantly lowers the risk of HIV infection from injectable drug use by 74% and sexual activity by 99%.^[10,11]^ However, several challenges persist in PrEP implementation, including determining the most effective delivery strategies for various demographics and understanding the social and behavioral impacts across different subpopulations, including women of reproductive age.^[12]^ These challenges highlight the importance of understanding the knowledge levels and attitudes toward PrEP among women of reproductive age in Tanzania.

PrEP presents a crucial opportunity to support ongoing public health initiatives aimed at eradicating HIV among women, advancing the goal of achieving HIV epidemic control and lowering the rate of mother-to-child transmission of HIV by avoiding HIV infection during pregnant and breast feeding.^[13,14]^ At present, most HIV infections in infants can be attributed to women who have recently been diagnosed with HIV, rather than those who are already on antiretroviral treatment.^[15]^ These show the importance of using PrEP to reduce mother-to-child HIV transmission among women of reproductive age.^[16]^ However, poor knowledge and low uptake of PrEP among women by below 40%^[17,18]^ present a significant barrier to achieving this public health initiative.

Other studies have produced contrasting results regarding the knowledge and attitudes toward PrEP. A survey conducted across 5 sub-Saharan African countries found that only 13.8% of the women exhibited knowledge and positive attitudes toward PrEP. Factors contributing to this knowledge include being over the age of 25 and having prior awareness of sexually transmitted infections (STIs).^[19]^ In the United States, similar research revealed a low level of PrEP knowledge among women, with only 0% to 33% reporting familiarity with the medication.^[9]^ A study conducted in Ghana reported the influence of socio-economic status on women’s knowledge and attitudes toward HIV-related services, including PrEP.^[20]^ Moreover, Tanzania ranks among the sub-Saharan African countries with the lowest levels of knowledge and positive attitudes toward PrEP, with only 5.6% of women being aware of it. This is in stark contrast to neighboring countries, such as Kenya (34.3%), Ghana (12.1%), Côte d’Ivoire (9.6%), and Burkina Faso (6.4%), which reported higher levels of awareness.^[19]^

Despite the WHO’s strong recommendation for oral PrEP as an effective HIV prevention strategy, women of reproductive age in Tanzania continue to face significant barriers to its adoption. As of 2022/2023, women in Tanzania exhibit a higher HIV incidence (0.24%) and prevalence (5.6%) than men, yet their PrEP utilization remains critically low. This disparity is particularly alarming given the proven efficacy of PrEP in reducing HIV transmission risk by up to 99% for sexual exposure.^[21,22]^ The persistent gap between the potential and actual use of PrEP among women of reproductive age suggests that the underlying complex factors related to knowledge, attitudes, social norms, and access remain poorly understood. Addressing this knowledge deficit is crucial for developing targeted interventions that can effectively improve PrEP uptake, ultimately reducing HIV vulnerability for women in Tanzania and potentially serving as a model for similar high-risk populations in sub-Saharan Africa (SSA). To assist in directing PrEP programs and helping Tanzania achieve the HIV epidemic by 2030, there is a need to understand the knowledge and attitude towards PrEP and its associated factors. Therefore, this study utilized nationally representative survey data to assess the factors associated with knowledge of and attitudes toward PrEP among women of reproductive age in Tanzania.

## 2. Methods

### 2.1. Data source, study area, and design

This study was an analytical cross-sectional survey that utilized secondary data from the 2022 Tanzania demographic health survey (TDHS), which conducts nationally representative population-based household surveys, typically every 5 years. The data were extracted in the individual file recode TZIR82FL. The Tanzania National Bureau of Statistics conducted the survey in collaboration with the Ministries of Tanzania Mainland and Zanzibar. Tanzania is a country in East Africa, covering an area of 945,087 square kilometers, including 61,000 square kilometers of inland water. The 2022 Tanzania Census reported that the country’s population is approximately 62 million.^[23]^

### 2.2. Population and sampling

Data for this study were obtained from the latest TDHS conducted between February 24, 2022, and July 21, 2022, across all regions in Tanzania. The target population for the 2022 TDHS includes women of reproductive age (15–49 years) across 32 administrative regions in Tanzania. A sampling frame is typically obtained at a national level. The country was stratified by geographic region and urban/rural areas, followed by 2-stage sampling to select a household to be surveyed. The first sampling step was to select a primary sampling unit (PSU) and then select a household. PSUs are survey clusters typically based on census enumeration areas. This was followed by selecting a fixed number of households to be surveyed using equal-probability systematic sampling. All women who had spent the night before the survey in the selected households were eligible to participate. A total of 15,254 women of reproductive age were interviewed and all were included in this study.

### 2.3. Variable measurements

#### 2.3.1. Dependent variable

The dependent variable in this study was women’s knowledge and attitudes towards HIV PrEP, a preventive measure against HIV infection. This study evaluated participants’ knowledge and attitudes as key outcome. Women were asked about their awareness and views on HIV PrEP, specifically whether they had never heard of it, had heard of it, approved of taking it daily, had heard of it but did not approve of taking it, or were unsure about approving it. For the purposes of this study, women who were familiar with HIV PrEP and expressed a positive attitude towards it were considered knowledgeable and had positive attitudes. These participants were recoded as “Yes = 1,” while those who did not meet these criteria were recoded as “No = 0.”^[19]^

#### 2.3.2. Independent variables

This current study examined explanatory variables based on available data.^[19]^ The variables were: age in years (15–24, 25–34, or 35–49), education level (no formal education, primary, secondary, or higher), marital status (never married, married, cohabiting, or separated/divorced), wealth index (poorest, poorer, middle, richer, or richest), ever tested for HIV (yes or no), ever heard of STI (yes or no), media exposure (yes as listening to the radio, reading the newspaper or watching television less than once a week or at least once a week or no if otherwise), parity (none, 1–3, ≥4), working status (working or not working), contraceptive use (yes or no), visited a health facility in the past 12 months (yes or no), place of residence (urban or rural), and geographical zones (Western, Northern, Central, Lake, Southern, Eastern, or Zanzibar).

### 2.4. Statistical analysis

To account for the complexity of the survey design, we applied individual sampling weights (v005/1,000,000), PSUs (clusters), and strata to adjust the cluster sampling design. Descriptive statistics were summarized using means, standard deviation for continuous variable, frequency, and proportion for categorical variables. All analyses were performed using STATA version 18.5 (STATA Corp, College Station). The Pearson chi-square test was used to compare the differences in the proportion of the knowledge and attitude to PrEP across participants characteristics.

We used a generalized logistic regression model with robust variance estimation to analyze the data. First, we performed a univariate analysis to estimate crude odds ratio for each explanatory variable’s association with the outcome variable. For variable selection in the multivariable analysis, we applied a *P*-value threshold of <.1 during the initial univariate screening to avoid prematurely excluding potentially important covariates. Since all our explanatory variables met this criterion, they were all considered for the subsequent multivariable analysis. To check for multicollinearity, which could affect the reliability of the multivariable model, we calculated the variance inflation factor and found a mean variance inflation factor of 4.32, suggesting no significant multicollinearity. Finally, we fitted a generalized multivariable logistic regression model, adjusting for potential confounders like respondent age, to estimate the adjusted odds ratio (AOR) and its corresponding 95% confidence interval (CI). The AOR and CI quantify the magnitude and strength of the associations, and statistical significance was set at *P* < .05.

## 3. Results

### 3.1. Demographic characteristics of study participants (women of reproductive age)

More than one-third (38.1%) were aged 15 to 24 years with an overall mean age of 29.3 years (standard deviation = 9.8). More than half (53.3%) had attained primary education and 60.7% were either married or living with a partner. The majority (69.3%) were exposed to the media and 64.3% were working. Less than one-third (30.9%) used contraceptives and 31.3% had more than 3 children. More than half (53.0%) had visited a health facility in the past 12 months and 78.1% had heard of STIs. There was a significant difference between knowledge of and attitude toward PrEP for all variables (*P* < .05), except for residence (Table 1).

**Table 1:** Sociodemographic characteristics and distribution of knowledge and attitude towards PrEP among women of reproductive age in Tanzania (N = 15,254).

Characteristics	N (%)	Knowledge and attitude to PrEP, n (%)		*P*-value
		Not knowledgeable with negative attitudes	Knowledgeable with positive attitudes	
Overall prevalence	–	14,399 (94.4)	854 (5.6)	–
Age group				
15–24	5 810 (38.1)	5 600 (96.4)	210 (3.6)	<.001
25–34	4 609 (30.2)	4 303 (93.4)	306 (6.7)	
35–49	4 835 (31.7)	4496 (93.0)	338 (7.0)	
Marital status				
Single	4 047 (26.5)	3898 (96.3)	149 (3.7)	<.001
Married/cohabiting	9 252 (60.7)	8685 (93.9)	567 (6.1)	
Separated/widowed	1 955 (12.8)	1815 (92.9)	140 (7.1)	
Education level				
No formal education	2 450 (16.1)	2353 (96.1)	97 (3.9)	.001
Primary	8 123 (53.3)	7683 (94.6)	440 (5.4)	
Secondary/higher	4 681 (30.7)	4362 (93.2)	319 (6.8)	
Media exposure				
No	4 690 (30.7)	4510 (96.1)	180 (3.9)	<.001
Yes	10,564 (69.3)	9889 (93.6)	675 (6.4)	
Parity				
None	3 874 (25.4)	3749 (96.8)	125 (3.2)	<.001
1–3	6 609 (43.3)	6178 (93.5)	430 (6.5)	
≥4	4 772 (31.3)	4471 (93.7)	301 (6.3)	
Working status				
Not working	5 452 (35.7)	5232 (96.0)	220 (4.0)	<.001
Working	9 802 (64.3)	9167 (93.5)	635 (6.4)	
Current on contraceptive use				
No	10,536 (69.1)	1005 (95.0)	531 (5.0)	.001
Yes	4718 (30.9)	4393 (93.1)	325 (6.9)	
Wealth index				
Poorest	2466 (16.2)	2388 (96.9)	78 (3.1)	<.001
Poorer	2578 (16.9)	2470 (95.8)	108 (4.2)	
Middle	2880 (18.9)	2720 (94.4)	160 (5.6)	
Rich	3359 (22.0)	3139 (93.4)	220 (6.5)	
Richest	3971 (26.0)	3681 (92.7)	290 (7.3)	
Visited a health facility in last 12 mo				
No	7167 (47.0)	6855 (95.6)	312 (4.5)	<.001
Yes	8087 (53.0)	7544 (93.3)	544 (6.7)	
Ever heard of a sexually transmitted infection				
No	3336 (21.9)	3232 (96.9)	104 (3.1)	<.001
Yes	11,918 (78.1)	11,167 (93.7)	751 (6.3)	
Ever tested for HIV				
No	2973 (19.5)	2902 (97.6)	72 (2.4)	<.001
Yes	12,281 (80.5)	11,497 (93.6)	784 (6.4)	
Residence				
Urban	5446 (35.7)	5107 (93.8)	340 (6.2)	.159
Rural	9808 (64.3)	9292 (94.7)	516 (5.3)	
Geographical zones				
Western	1268 (8.3)	1096 (86.4)	172 (13.6)	<.001
Northern	1733 (11.4)	1657 (95.6)	76 (4.4)	
Central	1573 (10.3)	1533 (97.5)	40 (2.5)	
Southern	3051 (20.0)	2983 (97.8)	68 (2.2)	
Lake	4454 (29.2)	4085 (91.7)	369 (8.3)	
Eastern	2657 (17.4)	2558 (96.3)	99 (3.7)	
Zanzibar	517 (3.4)	485 (93.8)	32 (6.2)	

HIV = human immunodeficiency virus, PrEP = pre-exposure prophylaxis.

### 3.2. Logistic regression analysis of factors associated with knowledge and attitude towards HIV PrEP among women of reproductive age

In the adjusted analysis, women aged 25 to 34 years (AOR = 1.47, 95% CI = 1.11–1.95) and those aged 35 to 49 years (AOR = 2.09, 95% CI = 1.52–2.88) had higher odds of good knowledge and positive attitudes toward PrEP compared to women aged 15 to 24 years. Women with media exposure had significantly higher odds of good knowledge and positive attitudes toward PrEP than their counterparts (AOR = 1.30, 95% CI = 1.24–1.83). Women in the richer quantiles (AOR = 2.01, 95% CI = 1.39–2.89) and the richest quantiles (AOR = 2.22, 95% CI = 1.47–3.36) had increased odds of having good knowledge and positive attitudes toward PrEP than those in the poorest quantile. Additionally, women who had visited a health facility in the past 12 months (AOR = 1.32, 95% CI = 1.13–1.54), had ever heard of STIs (AOR = 1.87, 95% CI = 1.48–2.38), and had ever been tested for HIV (AOR = 1.76, 95% CI = 1.33–2.33) had higher odds of good knowledge and positive attitudes toward PrEP than their counterparts (Table 2).

**Table 2:** Logistic regression analysis for factors associated with knowledge and attitude towards HIV PrEP among women of reproductive age in Tanzania (N = 15,254).

Characteristics	Crude	*P*-value	Adjusted	*P*-value
	OR (95% CI)		OR (95% CI)	
Age group				
15–24	Ref	–	Ref	–
25–34	1.90 (1.51–2.39)	<.001	1.47 (1.11–1.95)	.007
35–49	2.00 (1.60–2.51)	<.001	2.09 (1.52–2.88)	<.001
Marital status				
Single	Ref	–	Ref	–
Married/cohabiting	1.71 (1.37–2.16)	<.001	1.04 (0.85–1.44)	.833
Separated/widowed	2.02 (1.47–2.77)	<.001	1.15 (0.79–1.69)	.462
Education level				
No formal education	Ref	–	Ref	–
Primary	1.39 (1.06–1.82)	.018	1.28 (0.96–1.70)	.094
Secondary/higher	1.78 (1.35–2.34)	<.001	2.02 (1.44–2.84)	<.001
Media exposure				
No	Ref	–	Ref	–
Yes	1.70 (1.38–2.10)	<.001	1.30 (1.24–1.83)	.006
Parity				
None	Ref	–	Ref	–
1–3	2.09 (1.63–2.68)	<.001	1.16 (0.88–1.79)	.204
≥4	2.01 (1.56–2.61)	<.001	0.10 (0.65–1.53)	.983
Working status				
Not working	Ref	–	Ref	–
Working	1.65 (1.34–2.03)	<.001	1.31 (1.05–1.62)	.015
Current contraceptive use				
No	Ref	–	Ref	–
Yes	1.39 (1.16–1.68)	<.001	1.20 (0.97–1.47)	.092
Wealth index				
Poorest	Ref	–	Ref	–
Poorer	1.35 (0.98–1.86)	.069	1.18 (0.84–1.65)	.332
Middle	1.82 (1.33–2.49)	<.001	1.70 (1.21–2.40)	.002
Rich	2.16 (1.59–2.94)	<.001	2.01 (1.39–2.89)	<.001
Richest	2.43 (1.82–3.24)	<.001	2.22 (1.47–3.36)	<.001
Visited a health facility last 12 mo				
No	Ref	–	Ref	–
Yes	1.58 (1.32–1.89)	<.001	1.44 (1.18–1.75)	<.001
Ever heard of a sexually transmitted infection				
No	Ref	–	Ref	–
Yes	2.09 (1.56–2.78)	<.001	1.51 (1.11–2.04)	.009
Ever tested for HIV				
No	Ref	–	Ref	–
Yes	2.75 (2.03–3.73)	<.001	1.48 (1.02–2.15)	.037
Residence				
Urban	Ref	–	Ref	–
Rural	0.83 (0.69–1.01)	.057	1.16 (0.89–1.52)	.269
Geographical zones				
Western	6.05 (3.81–9.59)	<.001	8.86 (5.58–14.04)	<.001
Northern	1.77 (1.08–2.92)	.024	1.54 (0.93–2.55)	.090
Central	Ref	–	Ref	–
Southern	0.88 (0.54–1.43)	.602	0.81 (0.50–1.31)	.387
Lake	3.47 (2.21–5.45)	<.001	3.86 (2.46–6.05)	<.001
Eastern	1.48 (0.89–2.45)	.130	1.07 (0.64–1.78)	.793
Zanzibar	2.56 (1.60–4.10)	<.001	1.85 (1.13–3.02)	.014

CI = confidence intervals, HIV = human immunodeficiency virus, OR = odds ratio, Ref = reference category.

## 4. Discussion

Using nationally representative survey data of women from Tanzania, we found that the following factors were significantly associated with HIV-PrEP knowledge and attitudes among women of reproductive age: older age, those with secondary/higher education level, those with media exposure, those with a higher wealth index, those who visited a health facility in the last 12 months, those who had ever been tested for HIV, and those who had ever heard of a STI. Moreover, we found low knowledge and attitude toward HIV PrEP for HIV Infection at 5.6% compared to other studies across SSA: 34.3% for Kenya, 12.1% for Ghana, 9.6% for Cote d’Ivoire, 6.4% for Burkina Faso,^[19]^ and 10% for South Africa.^[24]^ The low knowledge and negative attitude towards PrEP in Tanzania, as reported in this study may be caused by different issues, including the Tanzania PrEP framework guidelines highlighting a few subpopulations eligible for PrEP Initiation, including female sex workers, men having sex with men (MSM), adolescent girls and younger women (AGYW), and discordant couples,^[25]^ and excluding other groups at substantial risk of HIV infection, including women of reproductive age. Although various social, cultural, behavioral, and structural factors may contribute to this high burden of low attitude and knowledge towards HIV PrEP,^[26]^ in terms of social factors, the inequality of access to other social services can lead to a loss of opportunities for HIV prevention among women. In terms of cultural factors, PrEP has the potential to create a sense of “false security” among users who may end up engaging in risky behavior.^[27]^ Therefore, the provision of PrEP to populations with substantial HIV risk should be recommended in PrEP guidelines rather than recommendation of PrEP provision to specific populations only.

In this study, we found that as age increases, the odds of HIV PrEP knowledge and attitude increases by 2.09 times for those aged 35 to 49 years and 1.47 times for those aged 25 to 34 years compared to those aged 15 to 24 years. A consistent finding was reported by a study conducted in Ghana,^[20]^ that AGYW had 11.3% and 31.5% lower knowledge of and negative attitudes towards PrEP, respectively, compared to other age groups. However, these findings are alarming issues among AGYW (15–24 years) because AGYW are reported to have a high HIV incidence of 0.33% compared to other age groups (0.32% for those aged 25–34 years, 0.20% for those aged 35–49 years) in Tanzania,^[8]^ and still the majority are unknowledgable with a negative attitude towards HIV PrEP. A possible explanation for this finding may be school-related factors, such as teachers’ negative attitudes, which discourage clinical access for HIV prevention, counseling, and treatment.^[28]^ The provision of integrated adolescent-friendly services with HIV prevention services across all clinics is important for increasing knowledge and attitude towards HIV PrEP.

Women who had media exposure were significantly associated with women’s knowledge and attitude towards HIV PrEP: women with media exposure had 1.30 times higher odds of being knowledgeable with a positive attitude toward HIV PrEP than those without media exposure. This finding is supported by different studies, including a meta-analysis study,^[29]^ highlighting that mass media interventions may help lower global HIV/AIDS disparities because of their reach and effectiveness across the population. Another study highlighted that print media and Internet websites are important sources of PrEP information across different geographical disparities.^[30]^ However, a national cross-sectional household-based survey conducted in Malawi^[31]^ highlighted media campaigns to increase awareness of HIV prevention across the community. These findings strongly support media engagement in increasing PrEP knowledge and attitudes toward HIV PrEP across geographical disparities.

In this study, those who visited health facilities within the last 12 months were significantly associated with HIV-PrEP knowledge and attitude, with higher odds of 1.44 times compared with those who did not visit health facilities within the last 12 months. This is consistent with the findings of a study conducted in Kenya^[27]^ and across SSA^[20]^ which reported that most health education is effectively provided in health facilities other than community settings. Despite this, a significant association was found with those who had heard of a STI having a higher odd of 1.51 times being knowledgeable and having a positive attitude toward HIV PrEP compared to those who had never heard of STIs. The mere association of this finding may be due to the majority of services including HIV testing and STI services are well integrated with PrEP education among individuals seeking those services, Strengthening Health services integration with HIV prevention is also recommended by the WHO.^[2]^ Furthermore, different PrEP programs should focus on the integration of services across health facilities, as they increase knowledge and attitudes among women of reproductive age.

In this study, we found that women with secondary/higher education level had 2.02 times higher odds of being knowledgeable and a positive attitude towards HIV PrEP compared to those with either primary or no formal education. Additionally, educated women in SSA are more likely to receive HIV PrEP due to their greater capacity to decide on their sexual activity and negotiate safe sex practices.^[26]^ Different studies have shown that women’s empowerment through the provision of education is strongly linked with their ability to ask their partners to use HIV preventions such as condoms, refuse sex, and make decisions about their sexual behavior.^[32]^ To understand why these interactions occur, it is important to consider the role of education and wealth in shaping individuals’ knowledge, perceptions, and attitudes towards HIV. Education may serve as a vital factor for disseminating accurate information regarding HIV prevention, transmission, and treatment. Education is important for separating truthful information regarding HIV prevention.^[33]^

Those who have undergone HIV testing have shown a positive association with HIV-PrEP knowledge and attitude compared to those who have never been tested for HIV. This is influenced by the fact that the majority of service points tend to provide integrated services, and HIV testing points provide opportunities for healthcare providers to educate and counsel women on HIV prevention, including the use of PrEP.^[34]^

Women with a good wealth index were found to have knowledge of and positive attitudes towards HIV PrEP, Consistent with a study conducted in Ghana, where the richest women were found to be knowledgeable with positive attitudes towards HIV-related issues.^[20]^ Likewise, women with a higher wealth index can have access to resources, opportunities, and better access to healthcare services, including HIV prevention, testing, and treatment, which can improve their understanding and attitudes towards the virus.^[26]^ Additionally, women with higher wealth indexes can easily access health information through different platforms, unlike those with low wealth indexes.

### 4.1. Strengths and limitations

This study has several strengths. First, it utilized a large sample size based on a nationally representative survey with probability sampling and weighted data. Second, the use of national survey data allows this study to offer valuable insights for policymakers in designing and implementing effective PrEP awareness and demand creation. However, this study had some limitations. Since the TDHS relies on self-reports, recall and social desirability biases may be present. Additionally, owing to the cross-sectional design of the survey, it was not possible to establish a temporal relationship.

## 5. Conclusion

The study uncovered several factors that are significantly associated with knowledge and positive attitudes towards HIV PrEP across Tanzania, using nationally representative survey data that will guide different PrEP interventions across the country. However, efforts should be made by PrEP programs focusing on subpopulations of women that were found to have no or low association with HIV-PrEP knowledge and attitude, including AGYW (15–24 years), those with no formal education/primary education, and those without media exposure. Additionally, strengthening PrEP education in health facilities service entry points, such as HIV testing points, STI screening, and patient departments (OPD) and community outreaches, are game changers to increase knowledge and altitude towards PrEP and use innovation such as media engagement.

### 5.1. Implications of the findings

These findings highlight the importance of addressing the barriers that prevent women from utilizing PrEP to slow the spread of HIV/AIDS. Efforts should be made by all stakeholders involved in HIV/AIDS prevention and control, to consider the factors identified in this study. Supporting economically disadvantaged women and enhancing HIV-PrEP education in the workplace and educational settings would be beneficial. Additionally, improving PrEP awareness, media, and health talks can foster a more positive attitude toward HIV PrEP among women of reproductive age. These efforts might contribute to better women’s health and help to reduce the prevalence of HIV/AIDS.

## Author contributions

**Conceptualization:** Patrick Mwanahapa, Elihuruma Eliufoo Stephano, Mtoro J. Mtoro.

**Data curation:** Patrick Mwanahapa, Mtoro J. Mtoro.

**Formal analysis:** Mtoro J. Mtoro.

**Investigation:** Patrick Mwanahapa, Elihuruma Eliufoo Stephano, Aidat Mugula, Mtoro J. Mtoro.

**Methodology:** Patrick Mwanahapa, Elihuruma Eliufoo Stephano, Mtoro J. Mtoro.

**Project administration:** Patrick Mwanahapa, Mtoro J. Mtoro.

**Resources:** Patrick Mwanahapa, Elihuruma Eliufoo Stephano, Aidat Mugula, Mtoro J. Mtoro.

**Supervision:** Patrick Mwanahapa, Elihuruma Eliufoo Stephano, Mtoro J. Mtoro.

**Validation:** Patrick Mwanahapa, Mtoro J. Mtoro.

**Visualization:** Patrick Mwanahapa, Mtoro J. Mtoro.

**Writing – original draft:** Patrick Mwanahapa, Elihuruma Eliufoo Stephano, Mtoro J. Mtoro.

**Writing – review & editing:** Patrick Mwanahapa, Elihuruma Eliufoo Stephano, Aidat Mugula, Mtoro J. Mtoro.
